# Doliroside A from *Dolichos falcata Klein* suppressing amyloid β-protein 42 fibrillogenesis: An insight at molecular level

**DOI:** 10.1371/journal.pone.0186590

**Published:** 2017-10-30

**Authors:** Dongpu Li, Hongfei Yu, Qinxiong Lin, Yun Liu

**Affiliations:** 1 Tianjin Medical University General Hospital, Tianjin, China; 2 Beijing Key Laboratory of Bioprocess, College of Life Science and Technology, Beijing University of Chemical Technology, Beijing, China; 3 Pharmaceutical College, South-Central University for Nationalities, Wuhan, China; Russian Academy of Medical Sciences, RUSSIAN FEDERATION

## Abstract

A bioactive chemical constituent, doliroside A, from Chinese traditional herbal medicine Dolichos falcata Klein was isolated, purified and identified by 60% ethanol extraction, thin layer chromatography (TLC), high performance liquid chromatography (HPLC) and nuclear magnetic resonance (NMR) spectroscopy. Molecular interaction mechanism between doliroside and amyloid β42 protein was evaluated by thioflavin T fluorescence (ThT), circular dichroism (CD), atomic force microscope (AFM), and differential scanning calorimeter (DSC) from the aspects of kinetics, secondary structure, morphology, and thermodynamics, respectively. Results show that the purity of doliroside A is 99.9% by HPLC, and its chemical structure is identified by ^1^H- and ^13^C-NMR. Doliroside A is observed to be concentration-dependent inhibiting the fibrillation of Aβ42 with the IC50 value of 26.57 ± 1.6 μM. CD and DSC results imply that doliroside A can bind to the nuclei and oligomers of Aβ42 to form a stable complex and suppress Aβ42 fibrillation. AFM images show that doliroside A, after bound to the nuclei and oligomers, redirect Aβ42 into off-pathway, amorphous oligomers. These findings not only provide a full insight into the molecular interaction mechanisms between Aβ42 and doliroside A, but also facilitate the development of new native anti-AD drug of doliroside A compound.

## Introduction

Alzheimer’s disease (AD) is the most common neurodegenerative disease with the characterization of progressive loss of memory, aphasia and agnosia [1] especially popular in developed countries [2]. One of the main causes of AD is the aggregation of amyloid β-protein (Aβ) from soluble random-coil into β-sheet-rich fibrils [3]. Rajasekhar et al. [4] reviewed the Aβ aggregation mechanism and its toxic effects, and summarized the different classes of molecules to inhibit amyloidogenic precursor protein processing, Aβ oligomerization or fibrillogenesis. On the basis of the numbers of amino acids in the backbone structure, Aβ protein contains several different species (Aβ39–43). Among them, amyloid β-protein 42 (Aβ42) fibrillogenesis has been widely considered as the most crucial key for the onset of AD due to its extremely severe neurotoxicity [5–7] A common therapeutic strategy against AD pathology is to prevent Aβ42 fibrillation [7]. Numerous studies have demonstrated that native small molecular compounds, such as (−)-epigallocatechin-3-gallate (EGCG) [8] [9], myricetin [10], resveratrol [11]), and tramiprosate [12] (Gervais et al., 2007), can suppress Aβ42 fibrillogenesis, which will form low toxicity aggregate structure.

Therefore, it is very urgent and important to search for more and more native active inhibitors against Aβ42 fibrillogenesis to prevent AD pathology. Recently, increasing interests have been focused on Chinese traditional herbal drugs and their active ingredients against AD pathology treatment in China. However, the molecular interaction mechanisms of Aβ42 and those bioactive chemical constituents from herbal drugs remain unclear to date.

*Dolichos falcata Klein*, a Chinese Dai ethnic medicine popularly known as “Tuoyeteng” in Yunnan province of China, has been widely used as a traditional herbal medicine to treat the fracture and beriberoid disease throughout the country, especially in countryside of China [13]. The voucher specimen of *Dolichos falcatus Klein* (No. 0610449, listed in Figure A in S1 File) has been kept in the Chinese Academy of Sciences Kunming Institute of Botany (Kunming, China). Since 2010, this plant is also called as *Dolichos tenuicaulis* (Baker) Craib, which is found in the Flora of China. It has been reported that *Dolichos falcata Klein* is popularly employed in medicinal preparations in China, such as “Yunnan Hongyao Jiaonang” in the market and “Damayao San” in clinical application, to treat fracture, rheumatoid arthritis, and soft tissue injuries by dispelling wind-evil and eliminating dampness, and activating blood flow and removing blood stasis according to the theory of Chinese medicine[13].

**Fig 1:**
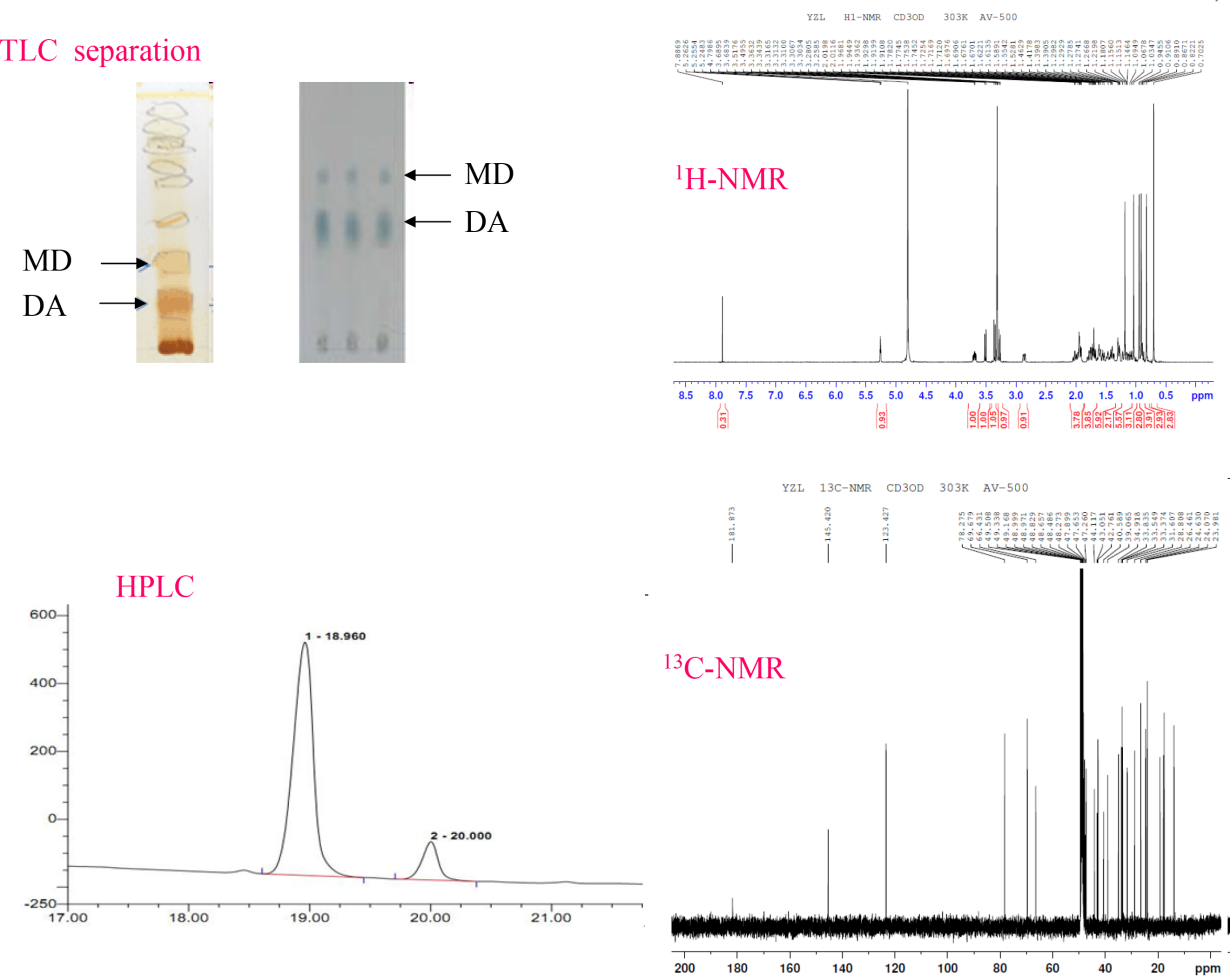
Two compounds, (1) medicagenicacid-3-O-β-D-glucopyranoside (MG), and (2) doliroside A (DA), separated by TLC (left up) and HPLC (left down) chromatography. The structure of doliroside A was identified by NMR (right).

Previous phytochemical studies have demonstrated that there are several biological active substances in the root and leaves of *Dolichos falcata Klein*, such as saponins, flavonoids, and triterpenes compounds, among which doliroside A is one of the major triterpene components to contribute to the anti-gouty arthritis effect [13] [14]. The content of doliroside A, a well-known active compound, in *Dolichos falcata Klein* plant was approximate 4% based on dried plant powder in the literature. However, in our present work, the HPLC fingerprintings are reported on the methanol extracts of *Dolichos falcata Klein* plant (seen in Fig 1). From HPLC curves, it is clearly obtained that the active compound, doliroside A (its retention time is 19.347 min in our work), is isolated and identified in our present work. The content of doliroside A is estimated to be 5.62%-7.5% based on dried plant powder dependence on the place of plant origin production. For instance, the content of doliroside A in this plant originating from Dali county in Yunnan province is about 5.62% based on dried plant powder, while the content doliroside A originating from Binchuan county in Yunnan province is about 7.5% based on dried plant powder.

*Dolichos falcata Klein* plant as a traditional medicine is popular among the people in rural areas in China, such as inflammation, diuresis, and wound healing [13][15–18]. Using traditional Chinese herbs to cure diseases in clinics have been acknowledged by people throughout the world, especially in China. And a lot of such articles have been available in the literature. For instance, Gao et al. [19] reviewed research progress on natural products from traditional Chinese medicine in treatment of Alzheimer's disease in 2013. Ruszkowski et al. [20] demonstrated that triterpenoids from traditional Chinese herbs could treat or slow the progression of neurodegenerative diseases such as Parkinson’s disease (PD), Alzheimer’s Disease (AD), Huntington’s disease. Liu et al. [21] reported that Xanthoceraside, a triterpenoid saponin extracted from Xanthoceras sorbifolia Bunge, showed positive effects in several Alzheimer’s disease. Du et al. [22] studied the molecular 108 mechanism underlying anti-inflammatory action of the triterpenoid saponin, Xanthoceraside. However, to date, no information has been available on the doliroside A for the treatment of AD pathology in the literature. In this case, the present study aims to extract and purify doliroside A from *Dolichos falcata Klein*, and assess the anti-AD pathology activities of this active compound from the molecular interaction mechanisms between doliroside A and Aβ42.

To meet above-mentioned challenge, doliroside A was firstly isolated, purified and identified using classical methods of methanol extraction, thin layer chromatography (TLC), high performance liquid chromatography (HPLC) and nuclear magnetic resonance (^1^H- and ^13^C-NMR) spectroscopies. Subsequently, the activities of doliroside A on anti-aggregating Aβ42 was evaluated to elucidate the molecular interactions between doliroside A and Aβ42 through thioflavin T (ThT) fluorescence spectroscopy, circular dichroism (CD) spectroscopy, atomic force microscope (AFM), and differential scanning calorimeter (DSC). The final results may support the wide utility as well as exploit a new clinical application in AD pathology of *Dolichos falcata Klein* in Chinese Dai ethnic medicine.

## Materials and methods

### Materials

The plant roots *of Dolichos falcata Klein* were bought from medicinal market in Yunnan province, China. The herbs were identified by Prof. Dingrong Wang, College of Pharmaceutical Sciences, South-Central University for Nationalities, China. Aβ42 peptide was purchased from GL Biochem (Shanghai) Ltd. (Shanghai, China) with the purity of higher than 95%. The amino acid sequence of Aβ42 is DAEFRHDSGYEVHHQKLVFFAEDVGSNKGAIIGLMVGGVVIA. Aβ42 was kept in –80°C fringe. Hexafluoroisopropanol (HFIP) with the purity of more than 99.5% was purchased from Sigma (St. Louis, MO, USA). Methanol (HPLC gradient grade) and butanol (AR grade) were purchase from China National Pharmaceutical Group Co. (Beijing, China). Other chemicals and agents were all of the analytic grades and bought from local markets in Beijing, China.

### Extraction, purification and identification of doliroside A from *Dolichos falcata Klein*

0.5 kg of the dried and powdered (sieved by 40 meshes) plant root was extracted with 60% methanol (solid/liquid ratio, 1:10 w/v) at 80°C for 2-h refluxing. Methanol crude extract (50 g) was extracted with butanol and then subjected to repeated chromatography on GF254 silica gel column and eluted with petroleum ether-ethyl acetate (50:1–1:1) and ethyl acetate-methanol (30:1–10:1) gradient solvent system. Two purified compounds, medicagenicacid-3-O-β-D-glucopyranoside and doliroside A, were obtained through recrystallization with methanol and acetone. The extraction flow chart of medicagenicacid-3-O-β-D-glucopyranoside and doliroside A was shown in Figure B in S1 File. The purification of this resulting compound was performed by HPLC methods. HPLC conditions: UltiMate 3000 High performance liquid chromatograph (HPLC); Nucleosil C18 (4.6mm×250mm×5μm) is used as chromatographic column; column temperature is 30°C; detection wavelength is 212 nm; Mobile phase is the mixture acetonitrile/ 0.1% acetic acid with the flow rate of 0.5mL/min. Finally, 200 mg white crystal compound was obtained, and its chemical structure was confirmed by ^1^H- and ^13^ C-NMR spectroscopies. NMR conditions: Bruker Avance II 400MHz nuclear magnetic resonance spectrometer; CD3OD is used as solvent.

### Content analysis of doliroside A by highperformance liquid chromatography (HPLC)

The purification of the isolated compound was performed on an UltiMate 3000 HPLC system (Shanghai, China). The content was analyzed using a Venusil MP C18 column (4.6 mm id × 250 mm, 5μm) with the UV detector wavelength at 212 nm. Column temperature was 30°C. The mobile phase used in separation included acetonitrile (solvent A) and 1% acetic acid (v/v) (solvent B). A gradient program was used as follows: 0–15 min, 10–50% A; 15–25 min, 50–35% A; 25–35 min, 35–100% A; 35–45 min, 100–10% A. The flow rate of mobile phase was set at 0.3 ml/min.

### Structural characterization of doliroside A by^1^H- and ^13^C-NMR measurement

Doliroside A structure was characterized by a Bruker Avance 500 Hz NMR spectrometer (Bruker, Germany) equipped with a 5-mm inverse-configuration probe with triple-axis-gradient capability at a field strength of 14.1T operating at 500.1, and 150.9MHz for^1^Hand ^13^C nuclei, respectively, at 303K with sample contained in CD_3_OD [23]. The chemical shifts of ^1^H and ^13^C nuclei are reported relative to tetramethylsilane (TMS) (*δ* = 0ppm for both ^1^H and ^13^C) using the solvent signals as secondary internal reference.

### *r* parameter determination

To facilitate the comparison of experimental data under various tested conditions, *r* representing the molar concentration ratio of doliroside A to Aâ42 was introduced in this work. It was calculated as follows:

$$r=\frac{\left[doliroside\;A\right]}{\left[A\beta 42\right]}$$

where [doliroside A] and [Aβ42] are the final molar concentrations of doliroside A and Aβ42 (μmol/L) in phosphate buffer solution (PBS) (100 mM, pH 7.4), respectively.

The final molar concentration of Aβ42 in PBS was 25 μmol/L, and the final molar concentrations of doliroside A in PBS were 2.5, 12.5, 25, 125, and 250 μmol/L, respectively. Therefore, in this work, the values of *r* were calculated at 0.1, 0.5, 1, 5, and 10, respectively.

### Fibrillation kinetics monitored by Thioflavin T (ThT) fluorescence spectroscopy

ThT fluorescence spectroscopy was employed to monitor the fibrillation kinetics of Aβ42 (25 μM in PBS, pH 7.4) in the absence and presence of dolisoride A at different concentrations (2.5 μM, 12.5 μM, 25 μM, 125 μM, and 250 μM in PBS, pH 7.4) at 37°C with the agitation of 200 rpm [24]. At appropriate intervals, aliquot of 200 μL samples was removed from the bulk solution and mixed with 2 mL of 20 μM ThT in PBS (pH 7.4). The solution was injected into a 1 cm-path length quartz cuvette and characterized by a Varian Cary Eclipse fluorescence spectrometer (California, USA). The excitation wavelength was 440 nm, and emission wavelength was 480 nm [21] The excitation and emission slits were both 10 nm. The scanning rate was 100 nm/min and the resolution was 1.0 nm.

### Thermodynamic parameter assessment by differential scanning calorimetry (DSC)

DSC curve was employed to investigate the transition midpoint temperature (*T*_m_) of Aβ42 in the absence and presence of dolisoride A at different concentrations (25 ìM, 125 ìM, and 250 ìM) during the fibrillation process. DSC measurement was performed on a Malvern MicroCal VP-DSC (MicroCal, Northampton, MA). The sample cell was loaded with the Aâ42 solution (25 ìM in PBS, pH 7.4), and the same concentration PBS was loaded into the reference cell as the blank control. Noticeably, the samples and control were degassed for 15 min immediately before DSC scanning. A buffer-buffer reference scan was subtracted from each sample scan prior to concentration normalization. Analysis conditions were: starting temperature 25°C, final temperature 90°C, scan rate 1.5°C/min, prescan 5 min, postscan 0 min, filtering period 10 s. DSC data were analyzed by MicroCal Origin Version 7.0.

### Conformation analysis by far-UV circular dichroism (CD) spectroscopy

The Aâ42 protein conformation variances were carried out in a JASCO J-810 automatic recording spectropolarimeter (Jasco Inc., Tokyo, Japan) controlled by the Jasco software. Briefly, Aâ42 (25 ìM) in the absence and presence of doliroside A (125 ìM) were incubated at 37°C for assigned periods. After co-incubation, the samples were centrifuged at 5,000 rpm for 15 min, and the supernatant was injected into a 1-mm path length quartz cuvette. A background CD spectrum of buffer solution was subtracted from the sample spectrum for baseline correction. Spectra were recorded under the conditions: a resolution of 0.5 nm, scanning rate of 100 nm/min, response time of 1 s, bandwidth of 2 nm, room temperature and the wavelength ranges from 250 to 190 nm.Morphology detection by atomic force microscope (AFM).

10 μL samples were pipetted onto freshly cleaved mica plate (1 cm × 1 cm) fixed onto a glass slide and incubated at room temperature for 3 min. The remaining saltsand loose deposits in the suspension were triplicate rinsed with ultrapure water (50 μL, Millipore) and then air-dried for a whole night (12 h). AFM images were obtained on a dimension FastScan AFM (Bruker, German) with FASTSCAN-A probe in ScanAsyst mode under ambient conditions. Scanning frequency was 1.95 Hz.

### Data statistical analyses

All values were expressed as the mean ± SD, and were analyzed by the Origin Pro 8.0 software (Origin Lab, USA).

## Results and discussion

### Structure identification of doliroside A from *Dolichos falcata Klein*

From the HPLC fingerprinting curves of methanol extracts from *Dolichos falcata Klein*, two components, medicagenicacid-3-O-β-D-glucopyr-anoside (MG, yield 0.5 g) and doliroside A (DA, yield 1.5 g), were isolated and purified from methanol extract by TLC and HPLC (Fig 1). The purities of two compounds were all tested to be higher than 99.5% by HPLC analysis. The chemical structures of the two purified compounds, DA and MG, are confirmed and identified by ^1^H- and ^13^ C-NMR. The NMR images of DA are shown in Fig 1, and the NMR curves of MG are depicted in Figure C in S1 File.

As shown in Fig 1, the chemical shifts of ^1^H and ^13^C nuclei are reported relative to TMS (*δ* = 0 ppm for both ^1^H and ^13^C) using the solvent signals as secondary internal references. The ^1^H-NMR (CD_3_OD, 500 MHz) spectrum of doliroside A revealed six methyl groups information (δ H 0.92, 0.97, 1.03, 1.28, 1.52, 1.97), and ^13^C-NMR (CD3OD, 500 MHz) showed the C chemical shifts δ. 43.49 (C-1), 68.76 (C-2), 83.33 (C-3), 51.04 (C-4), 51.10 (C-5), 19.9 (C-6), 32.06 (C-7), 40.00 (C-8), 47.49 (C-9), 35.76 (C-10), 22.59 (C-11), 121.46 (C-12), 143.79 (C-13), 40.80 (C-14), 27.06 (C-15), 21.44 (C-16), 45.70 (C-17), 41.42 (C-18), 45.40 (C-19), 30.33 (C-20), 33.30 (C-21), 32.78 (C-22), 178.56 (C-23), 13.35 (C-24), 16.04 (C-25), 16.72 (C-26), 25.57 (C-27), 199.75 (C-28), 32.14 (C-29), 23.07 (C-30), 73.68 (C-2'), 76.76 (C-3'), 70.05 (C-4'), 76.46 (C-5'), 61.02 (C-6').

**Fig 2:**
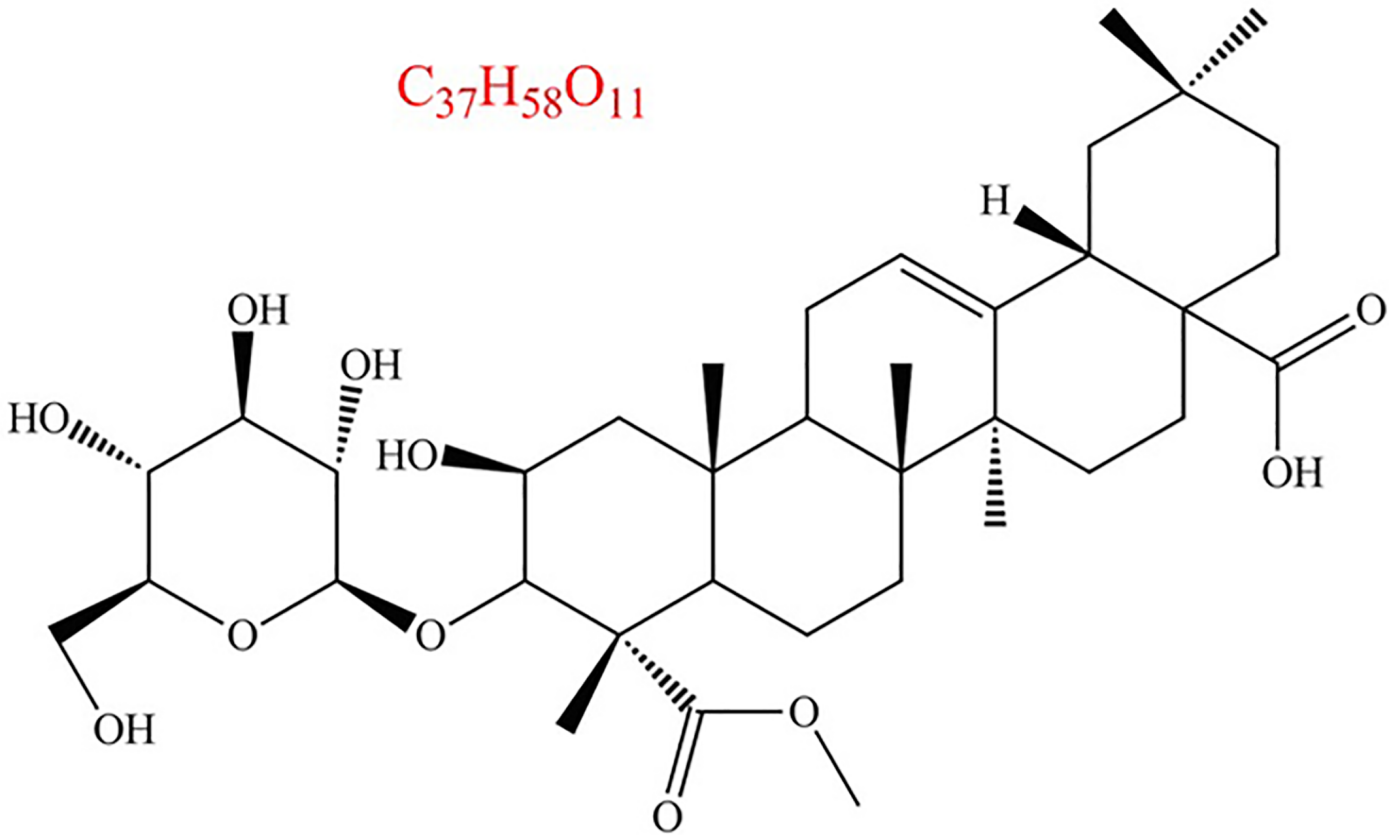
Chemical structural formula of doliroside A isolated from *Dolichos falcata Klein*.

From NMR data, the chemical structure of doliroside A, a major triterpenoid compound in *Dolichos falcate Klein*, was elucidated and shown in Fig 2. Chen and co-workers [13] demonstrated that doliroside A showed a significant effect on improving symptoms of acute gouty arthritis induced by monosodium urate crystals through inhibiting the production of pro-inflammatory cytokines. Wei and co-workers [14] reported that doliroside A attenuated monosodium urate crystals-induced inflammation by targeting nucleotide-binding domain and leucine-rich repeat containing protein3(NLRP3) inflammasome. It seems that doliroside A is an active compound against in flammation in jures [13][15–18]. However, no information has been available on the doliroside A for the treatment of AD pathology in the literature. In this case, the present work investigated the molecular interaction mechanism between Aβ42 and doliroside A to assess the anti-AD pathology activities of this active compound for the first time.

### Effect of doliroside A on the fibrillation kinetics ofAβ42

**Fig 3:**
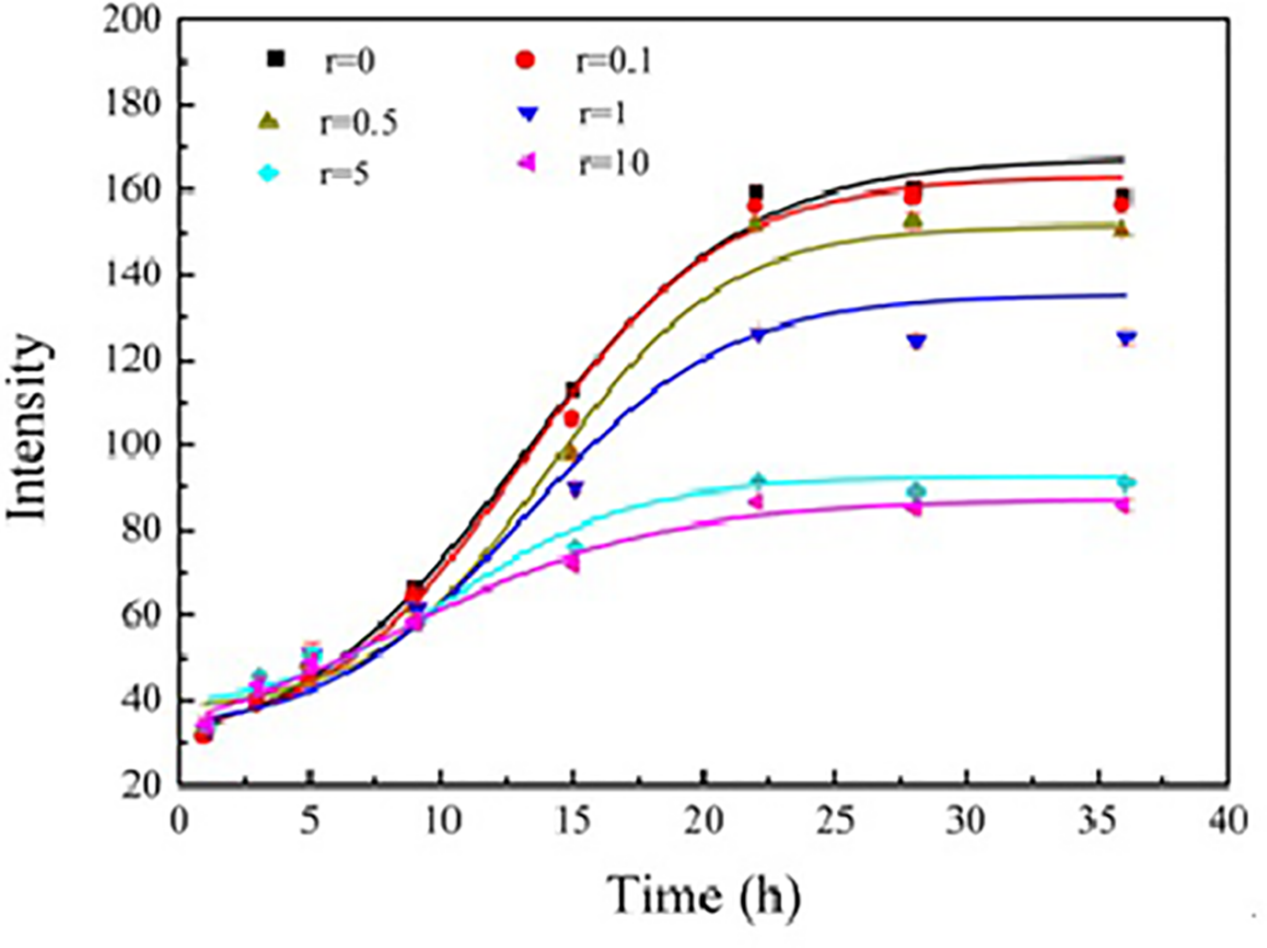
Kinetics of Aâ42 fibrillation in the absence and presence of doliroside A. The concentrations of Aâ42 and doliroside A were 25 μM and 2.5–250 μM, respectively. The *r* values derived from Eq. (1) were in the range of 0.1 to 10. Experiments were carried out in 100 mM PBS (pH7.4) at 37°C.

It has been reported that Aβ protofibrils and fibrils are rich in parallel β-sheets. ThT can be used as a probe for the assay of parallel β-sheet structure [25] (Blanchard et al., 2004), and the ThT fluorescence intensity at 480 nm (I480) is linearly related to the mass of Aβ42 fibrils [26]. Hence, the Aβ42 fibrillation kinetics in absence and presence of doliroside A could be quantified by I480 [27]. The results are shown in Fig 3.

As shown in Fig 3, the fibrillation of Aβ42 alone displayed a typical sigmoidal curve at the physiological condition (37°C and 100 mM PBS), and it contained three main stages of the fibrillation process: the lag phase of 0–5 h (1^st^ phase) where I480 showed slight changes, the exponential growth phase of 5–24 h (2^nd^ phase) where I480 increased exponentially with time, and the stable phase of > 24 h (3^rd^ phase) where I480 reached a plateau indicating the end of fibrillation process. The Aβ42 fibrillation process is a nucleation-dependent process, which sequentially contains three characteristic stages on a macroscopic level to form nanoscale amyloid fibrils [4] [28]: The 1^st^ phase is an initial slow nucleation stage, during which natively unfolded Aβ42 molecules form β-sheet-rich structure, assembling into initial nuclei. During the 2^nd^ phase, monomers and higher order oligomers elongate the nuclei into protofibrils. At the 3^rd^ phase, protofibrils further elongate into mature fibrils, leading to the end of the fibrillation.

Fig 3 also showed that doliroside A concentration-dependently inhibited the fibrillation of Aβ42. In other words, the ThT fluorescence intensity decreased with the increase of doliroside A concentrations from 2.5 to 250 μmol/L (*r* = 0.1–10). From these data, the 50% inhibition concentration (*IC*_50_) for doliroside A was calculated to be 26.57±1.6 μM. When *r* value was increased from 0.1 to 5, I480 declined significantly, while the further increase of *r* value from 5 to10 just caused slight decrease of I480.

### Effect of doliroside A on the secondary structure of Aβ42

**Fig 4:**
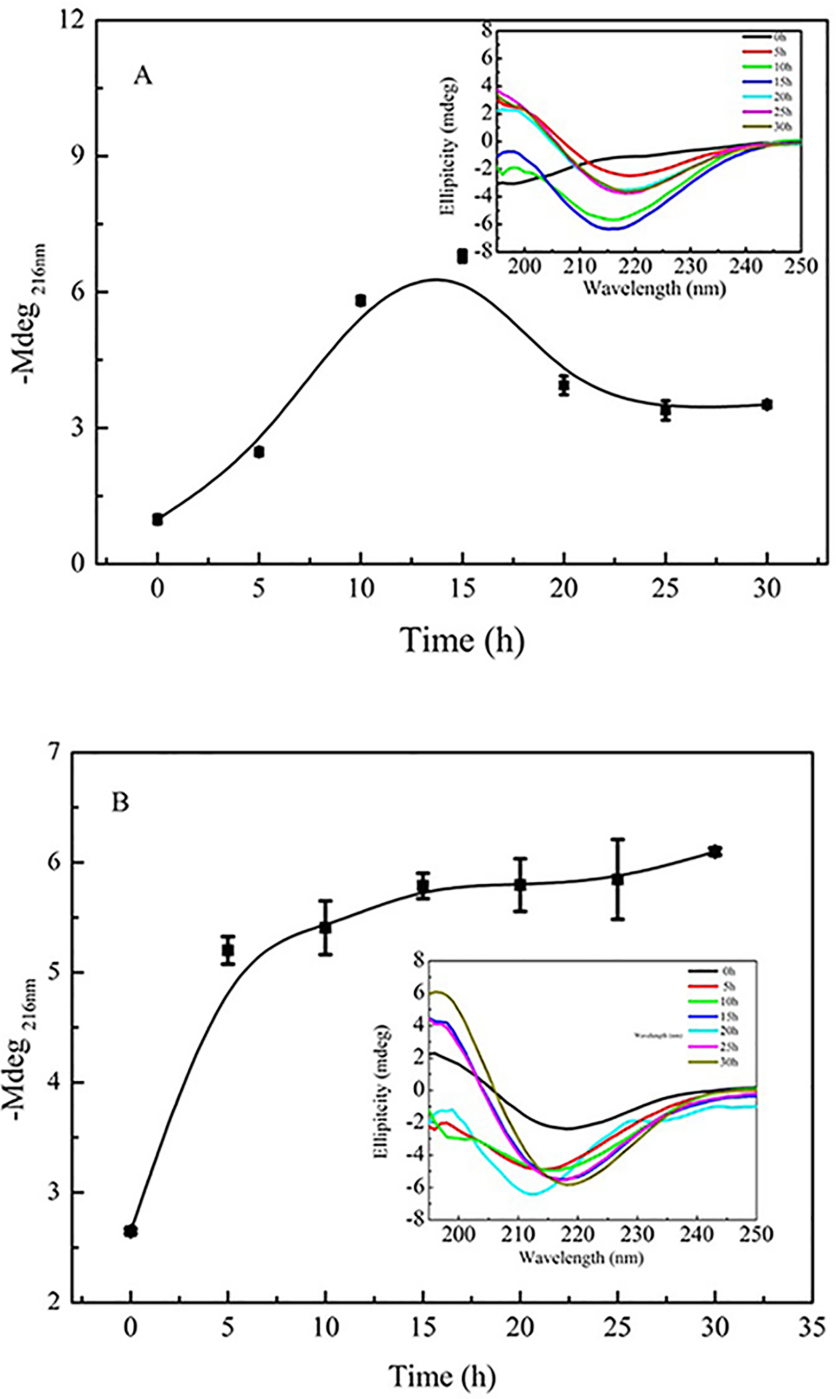
The intensity of CD at 216 nm (–Mdeg_216nm_) of Aâ_42_ (25 ìM) with the absence (A) and presence (B) of doliroside A (125 μM).The embedded graphs were the corresponding CD spectra at different incubation times.

The fibrillation of Aβ42 involves the conversion of unfolded Aβ42 molecules into β-sheet structure, the formation of highly ordered and β-sheet-rich nuclei, the elongation of nuclei to protofibrils, and the formation of insoluble β-sheet-rich mature fibrils [29]. Consequently, the change of β-sheet contents is a typical characteristic of the Aβ42 fibrillation [4]. So we further evaluated the effect of doliroside A on the secondary structure changes of Aβ42 during its fibrillation by CD spectroscopy. The CD spectra of Aβ42 without and with doliroside A were shown in the embedded graphs of Fig 4A and 4B.

As can be seen in the upper right corner of Fig 4A, the fibrillation of Aβ42 alone was accompanied by the conversion of secondary structure from random coil (characterized by the minimum at 200 nm) to β-sheet (characterized by the minimum at 216 nm). Since the β-sheet content is positively related to the intensity of CD at 216 nm (–Mdeg_216nm_), the negative Mdeg_216nm_ was plotted as a function of incubation time to visually see the changes of β-sheet contents during the Aβ42 fibrillation (Fig 4A). Prior to 15 h, the β-sheet contents increased with the increase of incubation time, which agreed well with the results in Fig 3 that unordered Aβ42 molecules gradually converted into β-sheet structure, nucleating, and elongating into protofibrils during 0 to 15 h. After 15 h, the β-sheet contents unexpectedly decreased and then plateaued at about 25 h. 15-25h, as shown in Fig 3, was the second half of the exponential growth phase (the 2^nd^ phase), at which protofibrils assembled into insoluble mature fibrils. So the fall of β-sheet content within this period mainly stemmed from the precipitation of insoluble fibrils during centrifugation before the CD assay (see the method section for detail information) [21]. This result was in consistence with that observed by Ruggeri et al. [30] during the study of influence of β-sheet content on the mechanical properties of Aβ42 fibrils. The decrease of β-sheet content was ascribed to the sedimentation of insoluble aggregates during the measurement [30].

When doliroside A was added into the Aβ42 solution in the initial lag phase, the negative Mdeg_216nm_ firstly increased rapidly at 0–5 h (the lag phase), and then slowly increased at 5–15 h. After 15 h, the Mdeg_216nm_ remained almost constant. It indicates that the β-sheet content became unchanged at this time (Fig 4B). It can be observed that the–Mdeg_216nm_ maximum of Aβ42 in the presence of doliroside A (6.10 ± 0.03) was lower than that of Aβ42 alone (6.79 ± 0.13), indicating that doliroside A prevented the increase of β-sheet content to some extent. Moreover, in the presence of doliroside A, the β-sheet content did not show decrease after 15 h, which suggested that doliroside A inhibited the formation of insoluble β-sheet-rich fibrils. Based on our experimental data, compound doliroside A inhibit Aβ fibril formation but do not inhibit oligomer formation. The results revealed that doliroside A compound appears to inhibit Abeta fibrillization by binding and stabilizing early oligomeric species, thus inhibiting the formation of larger aggregates. However, many studies have shown that early oligomeric species rich in beta-sheet could be even more toxic than fibrils to cells [4] [31] [32]. This cyctoxicity assay of Aβ oligomeric species is ongoing in our lab and will published in another paper in future.

### Effect of doliroside A on morphology of Aβ42 aggregates

**Fig 5:**
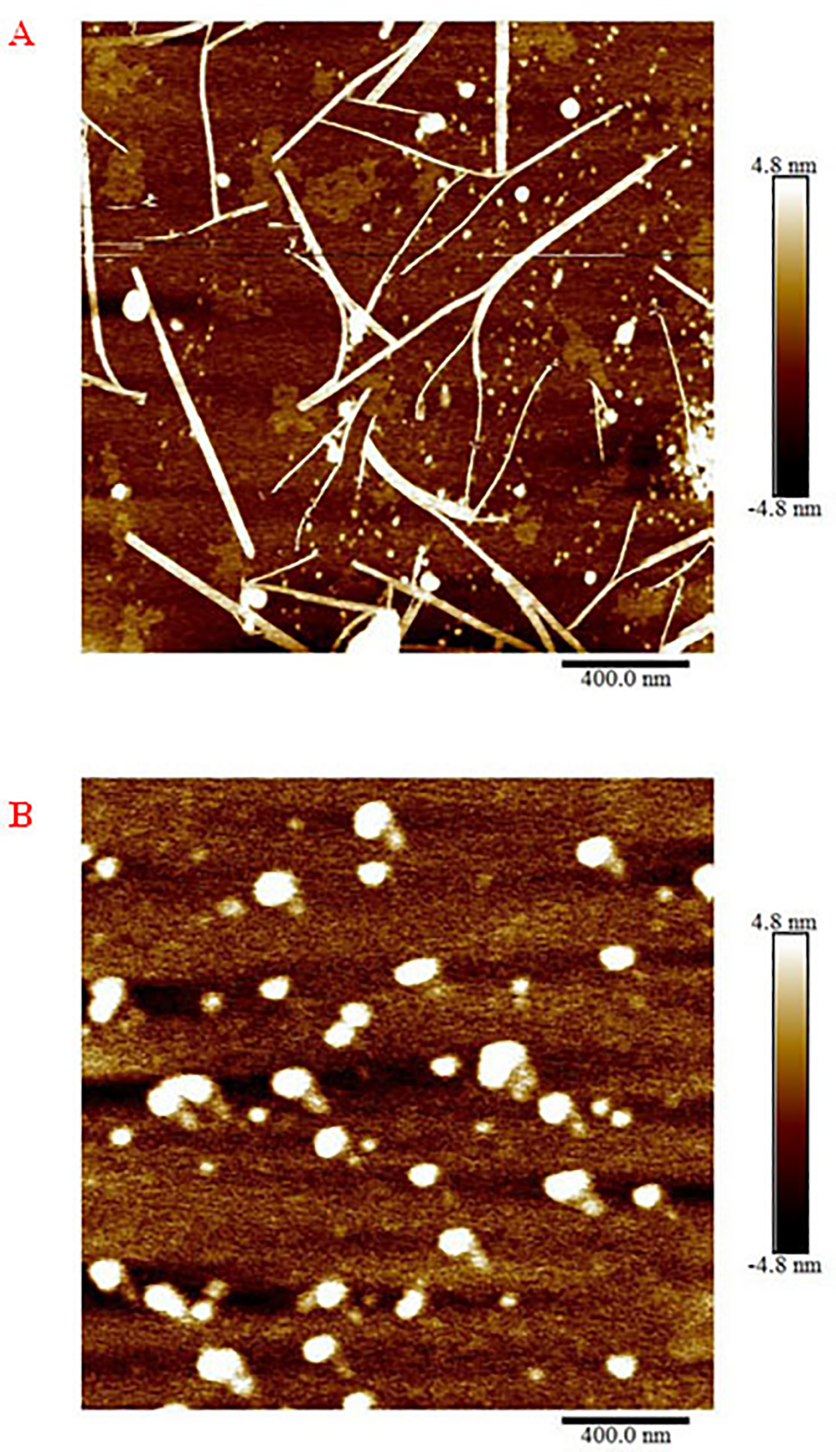
The morphology of Aβ42 aggregates in absence (A) and presence (B) of doliroside A added at stable phase (24 h) (the content of doliroside is 125 mΜ, *r* = 5).

The morphology of Aβ42 aggregates in the absence and presence of doliroside A was detected by AFM after 24-h incubation, and the AFM images were shown in Fig 5. Compared with the AFM image of Aβ42 alone in Fig 5A, amorphous aggregates, instead of fibrils, appeared at the equilibrium stage when adding doliroside A, revealing that the active compound doliroside A could suppress Aβ42 fibrillation and redirect it into off-pathway amorphous aggregates.

### Effect of doliroside A on the thermodynamic parameter (*T*_m_) of Aβ42

**Fig 6:**
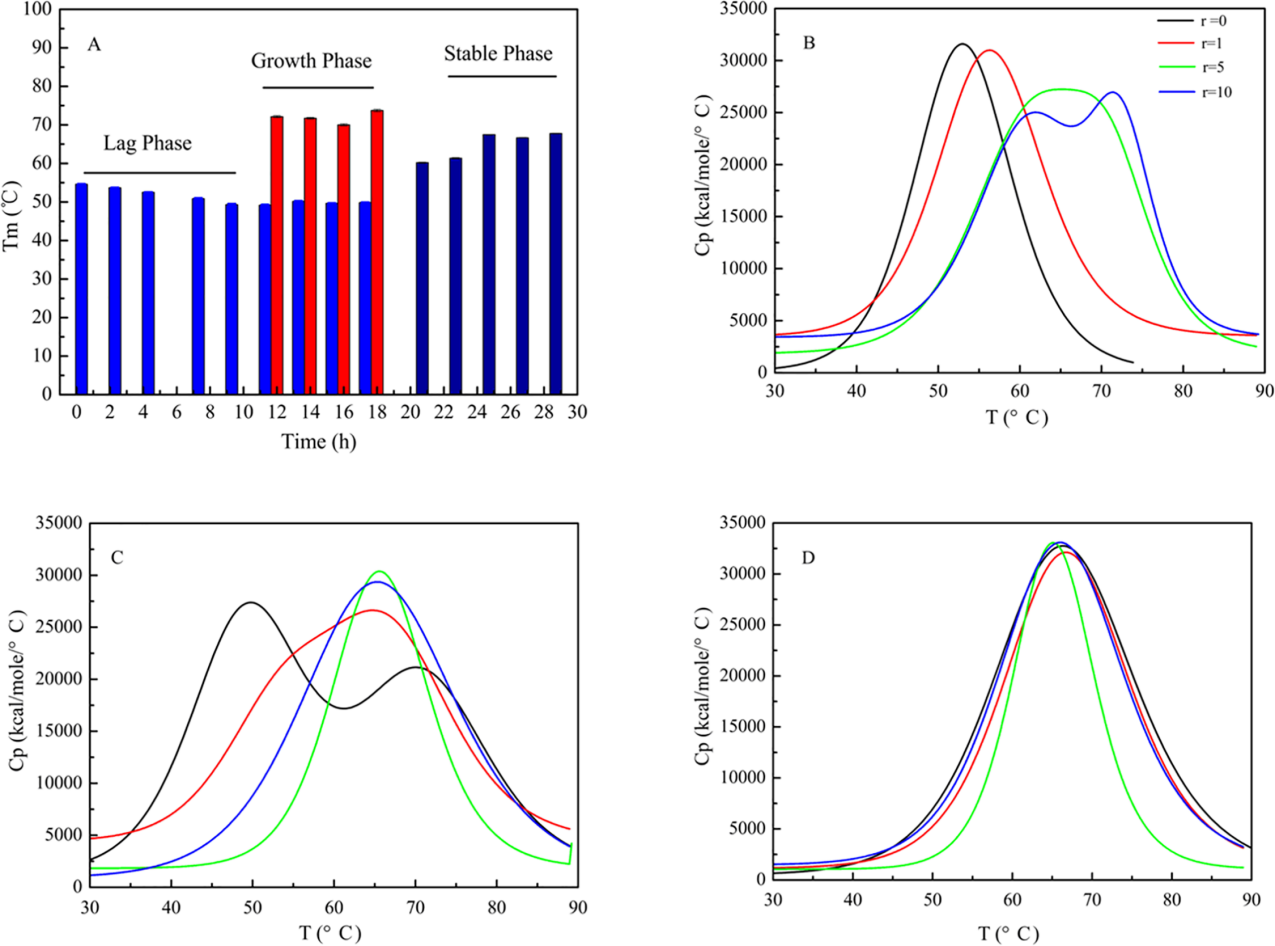
Effects of doliroside A concentration on thermodynamic parameter (*T*_m_) of Aβ42 for three steps of the Aβ42 fibrillation process when added at 3 h, 12 h and 24 h, respectively. Aβ42 concentration was 25 µM and doliroside A concentration could be calculated from the *r* values shown in the figure.

To address the effects of doliroside A on the thermostability of Aβ42 at different fibrillation phases, the DSC curves of Aβ42 were evaluated at different *r* values when doliroside A were added in the initial lag phase (0–10 h), growth phase (10–18 h), and stable phase (20-30h). The DSC curves were shown in Fig 6 and the transition midpoint temperature (*T*_m_) values picked from the DSC curves were listed in Table 1.

**Table 1:** The transition midpoint temperature (*T*_m_) calculated from DSC curves of Aβ42 in absence and presence of doliroside A.

*r* = [doliroside A]/[Aβ42]	Lag phase (1^st^ phase)		Growth phase (2^nd^ phase)		Stable phase (3^rd^ phase)
	*T*_m1_ (°C)	*T*_m2_ (°C)	*T*_m1_ (°C)	*T*_m2_ (°C)	*T*_m3_ (°C)
0	53.7±0.06	/	50.17±0.16	71.75±0.22	67.47±0.08
1	57.11±0.13	/	54.86±0.86	67.85±0.45	67.57±0.11
5	62.41±0.77	71.92±0.47	66.48±0.95	/	65.96±1.20
10	61.79±1.50	72.84±0.79	66.44±0.10	/	66.87±1.30

It was obviously observed from Fig 6A that there existed different *T*_m_ values at the three stages of Aβ42 fibrillation process. At 0–10 h, the 1^st^ phase, *T*_m1_ value decreased with the incubation time from 53.7 to 50.17°C, indicating that the thermostability of Aβ42 declined. Within this period, Aβ42 formed nuclei and oligomers according to the Th T intensity (Fig 3). The *T*_m1_ value decrease was probably ascribed to the fact that some early species of abeta gradually lost their stability as the aggregation process moved on. At 12–18 h, the 2^nd^ phase, another transition midpoint temperature, *T*_m2_, appeared and remained a constant value of 70°C within this stage. It suggested that more stable species were formed in the solution. At the fast growth phase, Aβ42 alone showed broad peak width and two *T*_m_ values (Fig 6C, black line), indicating that oligomers and protofibrils co-existed. At 20–30 h, the 3^rd^ phase, *T*_m1_ and *T*_m2_ merged into *T*_m3_ and gradually increased from 60 to 67°C. The combination of *T*_m1_ and *T*_m2_ indicated that most of the nuclei, oligomers, and protofibrils have formed relatively homogeneous mature fibrils at this stage. Moreover, the increase of *T*_m3_ value at 20–24 h was assigned to the secondary fibrillation and the formation of more stable and wider branched fibrils described in AFM images (Fig 5A).

By adding different concentrations of doliroside A into Aβ42 solution in the three steps of Aβ42 fibrillation process at 3 h, 12 h and 24 h, respectively, the impacts of doliroside A on the thermostability of Aβ42 were examined. From Fig 6B and Table 1, it can be found that, in the 1^st^ phase, *T*_m1_ value slightly increased from 53 to 57°C when adding doliroside A at *r* = 1. In cases of *r* = 5 and 10, another transition midpoint temperature (*T*_m2_) appeared, indicating that doliroside A stimulated Aβ42 molecules to form more stable complexes at the lag phase. Namely, doliroside A could redirect the fibrillation pathway of Aβ42 from the lag phase. When doliroside A was added at 2^nd^ phase of Aβ42 fibrillation, the *T*_m1_ and *T*_m2_ values merged and the peak width became narrower (Fig 6C) with increasing *r* values, suggesting that the homogeneity was increased by doliroside A. For instance, at *r* = 5 and 10, both *T*_m1_ and *T*_m2_ values disappeared and the *T*_m3_ value appeared which remained at 66.4°C. When doliroside A was added at the last phase, the *T*_m3_ value remained almost constant, the curves became shaper and narrower (r≥5), the *T*_m_ values were slightly lower than Aβ42 alone (seen in Fig 6A and Table 1).

### Interactions mechanism between doliroside A and Aβ42 protein

In this work, we for the first time reported that doliroside A extracted from *Dolichos falcata Klein* could concentration-dependently suppress the fibrillation of Aβ42 with the *IC*_50_ value of 26.57 ± 1.6 μM. Similar inhibitory behaviors were found with other native small molecules. For instance, Viet and co-workers [33] reported that dihydrochalcone from Eastern herbs and plants reduced the ThT fluorescence intensity of Aβ42 fibrils in a concentration-dependent manner (from 1 to 100 mM). The concentration of Aβ42 they used were 25 μM, which was the same as that in this study. In the presence of 1 mM Dihydrochalcone, the exponential growth phase was significantly decreased. Upon the addition of 100 mM dihydrochalcone, the I480 was decreased to ca. 66%. Shimmyo and co-workers [10] believed that myricetin could inhibit Aβ42 aggregation in a dose-dependent manner. When the myricetin concentration was 0.1 μM, weak inhibition activity was observed. In cases of myricetin concentration of 1μM and 10 μM, the inhibitory rate of myricetin on Aβ42 aggregation was enhanced from almost zero to 65.2 ± 5.1% [10].

The results in Fig 3 showed that doliroside A almost had no impact on the lag phase of Aβ42, while the *T*_m_ value in Fig 6B and Table 1 indicated that doliroside A could bind to Aβ42 and form more stable species when added in the lag phase. These phenomena implied that doliroside A mainly bound to the nuclei and oligomers of Aβ42 rather than monomers, which could not prolong the lag phase, but could significantly suppress the fast growth phase (Fig 3) [10] [23]. This deduction could also be supported by the CD result in Fig 4, which showed that the within the lag phase (0–5 h), the β-sheet content rapidly increased, while in the growth phase (5–24 h), the β-sheet content increased very slowly.

In our previously study, we investigated the interactions between Aβ42 protein and (–)-epigallocatechin 3-gallate (EGCG), the main component of green tea [21]. Although both doliroside A and EGCG redirected Aβ42 into off-pathway, amorphous aggregates (seen in Fig 5B), the interaction mechanisms were distinct. EGCG mainly bound to the unfolded Aβ42 molecules and prolonged the lag phase of Aβ42 fibrillation [21], while doliroside A bound to the nuclei and oligomers to form more stable complexes and slowed down the growth phase of Aβ42.

## Conclusions

In order to support the wide utility as well as exploit a new clinical application in AD pathology of *Dolichos falcata Klein* in Chinese Dai ethnic medicine, a triterpenoid active compound, doliroside A from this Chinese traditional herb was isolated, purified and identified by classic approaches, such as 60% methanol extraction, TLC, HPLC and NMR. The molecular interaction mechanisms between doliroside A and Aβ42 were elucidated for the first time via Th T fluorescence and CD spectroscopes, AFM, and DSC analyses. In conclusion, doliroside A showed the ability of suppressing Aβ42 aggregation in a concentration-dependent way with the *IC*_50_ value of 26.57 ± 1.6 μM. The inhibitory mechanisms of Aβ42 fibrillation was attributed to the fact that doliroside A could bound to the nuclei and ordered oligomers of Aβ42 to form more stable complexes, reducing the β-sheet content and redirecting it into off-pathway, amorphous aggregates. It is undoubted to speculate that doliroside A is a native promising inhibitor candidate against Aβ42 fibrillogenesis, which is helpful to take precaution against amyloid neurodegenerative diseases in future.

## Supporting information

S1 File **Figure A.)** The voucher specimen of *Dolichos falcatus Klein* (No. 0610449) in the Chinese Academy of Sciences Kunming Institute of Botany, Yunnan, China. **Figure B.)** The extraction flow chart of medicagenicacid-3-O-β-D-glucopyranoside and doliroside A from *Dolichos falcata Klein*. **Figure C.)** NMR analysis of medicagenicacid-3-O-β-D-glucopyranoside. NMR conditions: Bruker Avance II 400MHz nuclear magnetic resonance spectrometer (CD3OD is used as Solvent).
(DOC)

## Abbreviations

Aβ42: Amyloid β-protein 42
AD: Alzheimer’s disease
AFM: Atomic force microscope
CD: Circular dichroism
DA: Doliroside A
DSC: Differential scanning calorimeter
HPLC: High performance liquid chromatography
NMR: Nuclear magnetic resonance
PBS: Phosphate buffer solution
ThT: Thioflavin T
TLC: Thin layer chromatography
Tm: Thermodynamic parameter
