# Supplementary material for: Doliroside A from Dolichos falcata Klein suppressing amyloid β-protein 42 fibrillogenesis: An insight at molecular level
Source: PLoS One. 2017 Oct 30;12(10):e0186590. doi: 10.1371/journal.pone.0186590 (PMC5662078; doi:10.1371/journal.pone.0186590)

**Supplement Information**

**Figure A.** The voucher specimen of *Dolichos falcatus Klein* (No. 0610449) in the Chinese Academy of Sciences Kunming Institute of Botany, Yunnan, China.

**Figure B** The extraction flow chart of medicagenicacid-3-O-β-D-glucopyranoside and doliroside A from *Dolichos falcata Klein.*

**Figure C** NMR analysis of medicagenicacid-3-O-β-D-glucopyranoside. NMR conditions: Bruker Avance II 400MHz nuclear magnetic resonance spectrometer (CD3OD is used as Solvent).

**Fig. A**


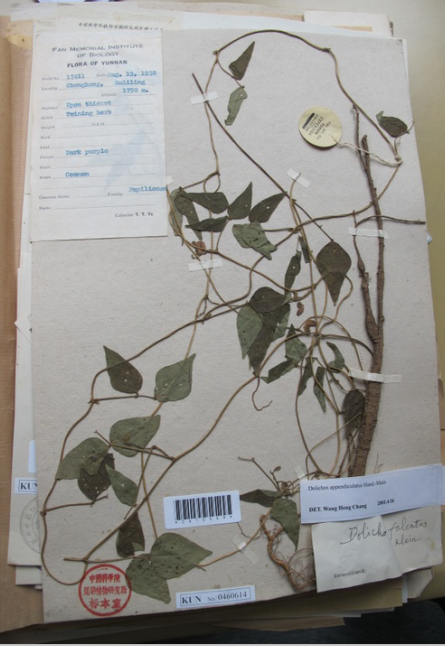

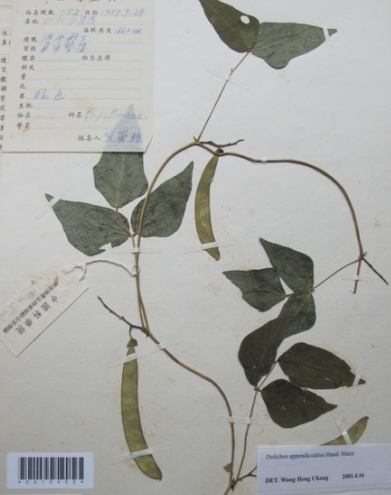


**Fig. B**


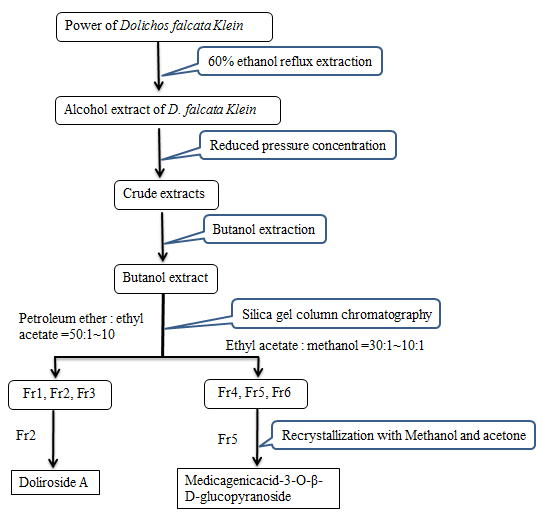


**Fig. C**


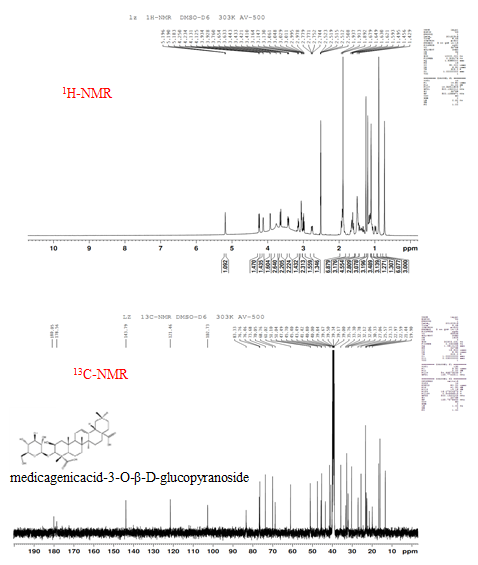

Supplement: S1 File — Figure A.) The voucher specimen of Dolichos falcatus Klein (No. 0610449) in the Chinese Academy of Sciences Kunming Institute of Botany, Yunnan, China. Figure B.) The extraction flow chart of medicagenicacid-3-O-β-D-glucopyranoside and doliroside A from Dolichos falcata Klein. Figure C.) NMR analysis of medicagenicacid-3-O-β-D-glucopyranoside. NMR conditions: Bruker Avance II 400MHz nuclear magnetic resonance spectrometer (CD3OD is used as Solvent). (DOC) [file pone.0186590.s001.doc]
